# Neurorescue Effects of Frondoside A and Ginsenoside Rg3 in *C. elegans* Model of Parkinson’s Disease

**DOI:** 10.3390/molecules26164843

**Published:** 2021-08-10

**Authors:** Pawanrat Chalorak, Tanatcha Sanguanphun, Tanapol Limboonreung, Krai Meemon

**Affiliations:** 1 Department of Anatomy, Faculty of Science, Mahidol University, Ratchathewi, Bangkok 10400, Thailand; airpawanratch@gmail.com (P.C.); tanatchasan.pun@gmail.com (T.S.); 2 Faculty of Dentistry, King Mongkut’s Institute of Technology Ladkrabang, Ladkrabang, Bangkok 10520, Thailand; tanapol.li@kmitl.ac.th

**Keywords:** Parkinson’s disease, dopaminergic neurons, neurodegeneration, α-Synuclein, frondoside A, ginsenoside Rg3, *Caenorhabditis elegans*

## Abstract

Parkinson’s disease (PD) is a currently incurable neurodegenerative disorder characterized by the loss of dopaminergic (DAergic) neurons in the substantia nigra pars compacta and α-synuclein aggregation. Accumulated evidence indicates that the saponins, especially from ginseng, have neuroprotective effects against neurodegenerative disorders. Interestingly, saponin can also be found in marine organisms such as the sea cucumber, but little is known about its effect in neurodegenerative disease, including PD. In this study, we investigated the anti-Parkinson effects of frondoside A (FA) from *Cucumaria frondosa* and ginsenoside Rg3 (Rg3) from *Panax notoginseng* in *C. elegans* PD model. Both saponins were tested for toxicity and optimal concentration by food clearance assay and used to treat 6-OHDA-induced BZ555 and transgenic α-synuclein NL5901 strains in *C. elegans*. Treatment with FA and Rg3 significantly attenuated DAergic neurodegeneration induced by 6-OHDA in BZ555 strain, improved basal slowing rate, and prolonged lifespan in the 6-OHDA-induced wild-type strain with downregulation of the apoptosis mediators, *egl-1* and *ced-3*, and upregulation of *sod-3* and *cat-2*. Interestingly, only FA reduced α-synuclein aggregation, rescued lifespan in NL5901, and upregulated the protein degradation regulators, including *ubh-4*, *hsf-1*, *hsp-16.1* and *hsp-16.2*. This study indicates that both FA and Rg3 possess beneficial effects in rescuing DAergic neurodegeneration in the 6-OHDA-induced *C. elegans* model through suppressing apoptosis mediators and stimulating antioxidant enzymes. In addition, FA could attenuate α-synuclein aggregation through the protein degradation process.

## 1. Introduction

Parkinson’s disease (PD) is the progressive neurodegenerative disease associated with loss of dopaminergic (DAergic) neurons in the substantia nigra leading to tremors, rigidity, slow movement, and postural instability [1]. Moreover, the histopathological study has identified Lewy’s body as a pathological hallmark of PD. The Lewy’s body is an inclusion body formed by the aggregation of α-synuclein and ubiquitinated proteins, suggesting the impairment of protein degradation pathways as a contributing factor to cell death [2]. Unfortunately, PD is an incurable disease, and only palliative care is available for PD patients [3]. Therefore, searching for suitable therapeutic strategies that could improve or slow down the disease progression is an urgent and necessary issue.

**Figure 1:**
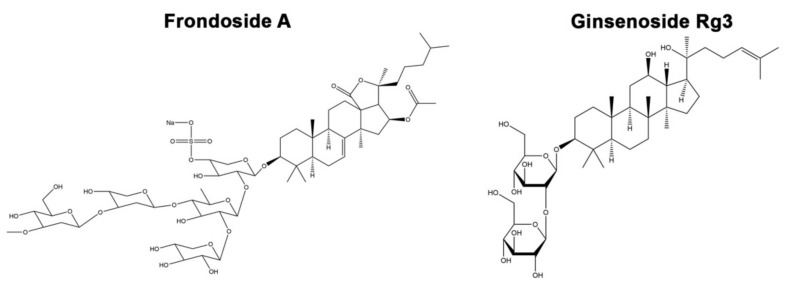
Structure of frondoside A (FA) and ginsenoside Rg3 (Rg3). (Drawn by Chemdraw).

Saponin is a group of compounds consisting of a hydrophobic aglycone backbone and hydrophilic sugar molecules, rendering it soluble in polar and nonpolar solvents [4]. This amphipathic property of saponin has drawn pharmacological attention to this group of compounds because it can cause diverse cellular activities by known pathways, including pore formation, direct membrane lysis, permeabilization of membrane, induced raft activity and direct protein binding [5]. Interestingly, various naturally occurring saponins have been reported to bind with the pathogenic proteins and cause an inhibitory aggregation effect, expected to solve protein aggregation-induced neurodegeneration disorders such as Huntington’s disease, Alzheimer’s disease, and Parkinson’s disease [6]. Ginsenoside Rg1, a plant triterpene saponin found in *Panax ginseng*, displayed protective effects in PC12 cell and mouse PD models by modulating antiapoptotic protein Bcl-2 and iNOS expressions [7]. Another triterpene saponin from *P. ginseng*, ginsenoside Rg2, also displayed protective effects in a cellular PD model [8]. Ginsenoside Rg3 (Rg3) is steroidal saponin extracted from *P. ginseng* and recently identified (Figure 1). It has been reported to improve mitochondrial dysfunction by regulating the abnormality in the energy metabolism in the Alzheimer’s disease mouse model [9]. The study in hippocampal neurons revealed that Rg3 antagonizes NMDA receptors, consequently reducing neuronal excitotoxicity [10]. Interestingly, the study in the prion protein-induced neurotoxicity model revealed that Rg3 could provoke the autophagy flux to resolve the neurotoxicity, suggesting its beneficial effect in proteotoxicity-related diseases, such as PD [11]. However, the effect of Rg3 relevant to PD has not been elucidated yet.

Natural saponins can also be found in marine invertebrates. Frondoside A (FA; Figure 1) is a bioactive compound found in sea cucumber *Cucumaria frondosa*, which has been used as a traditional remedy to cure several diseases. Previously, FA could inhibit proliferation of pancreatic cancer cells in the xenograft model [12]. Moreover, the anticancer activities of FA have been studied in several cancer cell types, including breast cancer [13], leukemia [14], and lung cancer [15]. Nevertheless, FA has rescued the amyloid β-induced proteotoxicity in the *C. elegans* model of Alzheimer’s disease, suggesting the protective effect of FA in the proteinopathic neurodegenerative disorders such as PD [16]. Therefore, we hypothesize that FA and Rg3 may attenuate DAergic neurodegeneration and α-synuclein aggregation, the major hallmarks of PD.

Testing the candidate therapeutic compounds in vertebrate disease models is a time-consuming and costly experimental study. *Caenorhabditis elegans* is an animal model that affords several advantages and is widely accepted in PD studies [17]. *C. elegans* has eight DAergic neurons, which are structurally and functionally similar to those of humans. The eight DAergic neurons consist of four anterior cephalic neurons (CEPs), two anterior deirid neurons (ADEs), and two posterior deirid neurons (PDEs). In this study, we employed the BZ555 and NL5901 *C. elegans* models to determine the anti-Parkinson effects of FA and Rg3. BZ555 is the strain that specifically tagged *C. elegans dat-1* promoter (dopamine transporter gene) with a green fluorescent protein (GFP) localizing the DAergic neurons. Thus, selective DAergic neurodegeneration can be induced by administering neurotoxins such as 6-hydroxydopamine (6-OHDA). Moreover, transgenic strain NL5901 expresses human α-synuclein fused with yellow fluorescent protein (YFP) in the *unc-54* promoter, thus α-synuclein accumulation is localized in muscle cells. Therefore, we used *C. elegans* as a model to evaluate the neurorescue effects of FA and Rg3 on PD and to investigate their potential mechanism of action.

## 2. Results

### 2.1. Determining Dose Range and Toxicity of FA and Rg3 on C. elegans by the Food Clearance Assay

**Figure 2:**
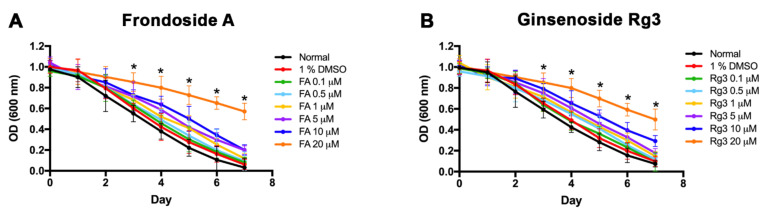
Food clearance assay of *C. elegans* treated with 0–20 μM FA (**A**) and Rg3 (**B**). The asterisk (*) indicates a significant difference between the compound-treated worms compared with 1% DMSO-treated worms (*p* < 0.01). The experiment was performed independently at least three times (number of worms = 30 animals/group per replicate).

To determine the toxicity of FA and Rg3 on growth and reproduction in the *C. elegans* model, the food clearance assay was performed. Various doses of FA and Rg3 (0.1, 0.5, 1, 5, 10 and 20 μM) were examined by measuring the OD600 value of *E. coli* suspension in *C. elegans* cultured with FA and Rg3. The OD600 value in the control experiment, only 1% dimethyl sulfoxide (DMSO) treatment, dramatically reduced at 4 days in wild-type N2 strain (Figure 2). The curve of the OD600 indicated that DMSO at a concentration of 1% has no effect on the food clearance. Moreover, the OD600 value also decreased in the FA (Figure 2A) and Rg3 treatments at 0–10 μM with no significant difference from the control group in all strains (Figure 2B). These results indicated that 0–10 μM FA and Rg3 have no toxicity in *C. elegans*. However, worms exposed to 20 μM FA or Rg3 showed a significant delay in bacterial clearance, indicating toxicity of these compounds at high doses. Therefore, FA and Rg3 at doses of 0.1, 0.5, 1, 5, and 10 μM were selected for use in the subsequent experiments.

### 2.2. FA and Rg3 Rescue the 6-OHDA-Induced DAergic Neurodegeneration in C. elegans

**Figure 3:**
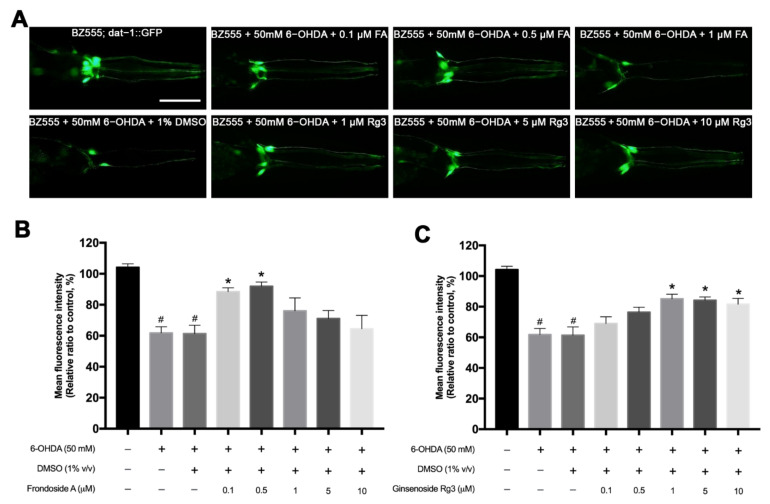
Effects of FA and Rg3 on restoration of 6-OHDA-induced DAergic neurodegeneration in *C. elegans* BZ555 strain. (**A**) GFP expression pattern in DAergic neurons of normal BZ555 strain, BZ555 exposed to 50 mM 6-OHDA, and BZ555 exposed to 50 mM 6-OHDA and treated with FA (0.1, 0.5, 1 μM) or Rg3 (1, 5, 10 μM). Scale bar, 50 μm. (**B**,**C**) Graphical representation for MFI of GFP expression in DAergic neurons treated with FA (**B**) and Rg3 (**C**) as measured by using ImageJ software. The hash (#) indicates a significant difference between 6-OHDA-induced and uninduced worms (*p* < 0.05). The asterisk (*) indicates a significant difference between the 6-OHDA-induced control worms and the FA/6-OHDA- or Rg3/6-OHDA-treated worms (*p* < 0.05). The experiment was performed independently at least three times (number of worms = 40–50 animals/group per replicate).

The morphological patterns of DAergic neurons in BZ555 *C. elegans* were shown by the green fluorescent protein expression in all six intact DAergic neurons, including four CEPs and two ADEs (Figure 3A). The selective degeneration of the DAergic neurons was achieved when exposed to 50 mM 6-OHDA. The exposure of 50 mM 6-OHDA in worms showed damage in the cell bodies of CEPs and ADEs and fragmented processes with significantly reduced relative mean fluorescence intensity (MFI) to 60% compared with the nonexposed worms (*p* < 0.05) (Figure 3A,B). The 6-OHDA/DMSO-induced group did not show a different loss of DAergic neurons and MFI compared with the 6-OHDA-induced only worms. In the treatment group, the microscopic results showed that 0.1, 0.5 μM of FA and 1, 5, 10 μM of Rg3 had the higher GFP intensities compared with the control 6-OHDA/DMSO-induced group (Figure 3A). The MFI of the worms treated with 0.1 and 0.5 μM FA significantly increased to 88.45% and 91.53%, respectively, (*p* < 0.05) (Figure 3B). Likewise, the worms treated with 1, 5 and 10 μM Rg3 exhibited significantly increased GFP expression to 85.15%, 84.24% and 81.58%, respectively (*p* < 0.05) (Figure 3C). In contrast, worms treated with 1, 5, 10 μM of FA and 0.1, 0.5 μM of Rg3 slightly increased the MFI with no significant differences from the controls (Figure 3B,C). In addition, FA showed a relative trend of toxicity at higher doses than 10 μM.

### 2.3. FA and Rg3 Recover the Basal Slowing Rate in 6-OHDA-Induced C. elegans 

**Figure 4:**
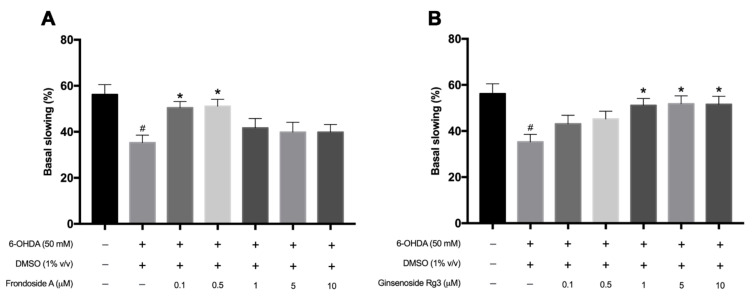
Graphical representations for the basal slowing rate in 6-OHDA-induced N2 *C. elegans* treated with FA (**A**) and Rg3 (**B**). The hash (#) indicates a significant difference between 6-OHDA-induced and uninduced worms (*p* < 0.05). The asterisk (*) indicates a significant difference between the 6-OHDA-induced control worms and the FA/6-OHDA- or Rg3/6-OHDA-treated worms (*p* < 0.05). The experiment was performed independently at least three times (number of worms = 40–50 animals/group per replicate).

Since 6-OHDA could cause the selective degeneration of DAergic neurons, we next examined dopamine-dependent behavior which is the basal slowing response. Previous studies showed that worms lacking dopamine, *cat-2* mutant, displayed the basal slowing deficits [18]. Similar to a previous study, 6-OHDA exposure significantly decreased the basal slowing rate when compared with normal worms (*p* < 0.05) (Figure 4A). Interestingly, treatment with FA (0.1, 0.5 μM) and Rg3 (1, 5, 10 μM) after 6-OHDA exposure showed a significant increase in the basal slowing rate compared with the 6-OHDA-induced only worms (*p* < 0.05) (Figure 4A,B). In contrast, worms treated with 1, 5, 10 μM of FA and 0.1, 0.5 μM of Rg3 extracts slightly increased the basal slowing rate with no significant difference. Taken together, these results indicated that FA (0.1, 0.5 μM) and Rg3 (1, 5, 10 μM) could rescue the degenerated DAergic neurons and ameliorate the DAergic functions caused by 6-OHDA exposure.

### 2.4. FA Reduces α-Synuclein Accumulation in Transgenic NL5901 C. elegans 

**Figure 5:**
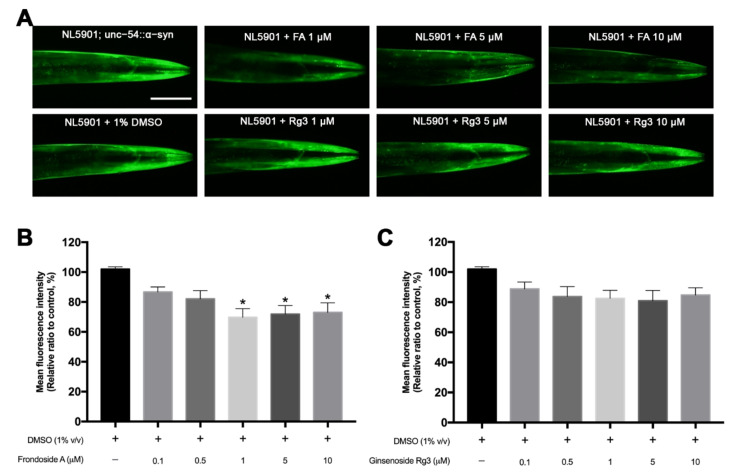
Effects of FA and Rg3 on reduction of α-synuclein accumulation in *C. elegans* NL5901 strain. (**A**) YFP expression of the α-synuclein accumulation in muscles of normal NL5901, NL5901 treated with various doses of FA or Rg3. Scale bar, 50 μm. (**B**,**C**) Graphical representations for MFI of YFP expression of α-synuclein accumulation treated with FA (**B**) and Rg3 (**C**) as measured by using ImageJ software. The asterisk (*) indicates a significant difference between the control DMSO-treated worms and the FA- or Rg3-treated worms (*p* < 0.05). The experiment was performed independently at least three times (number of worms = 40–50 animals/group per replicate).

To determine whether FA and Rg3 have an effect on α-synuclein degradation, we used the *C. elegans* NL5901 strain which expresses human α-synuclein fused with YFP under a control of *unc-54* promoter. As shown in the untreated NL5901, worms highly expressed the aggregation of α-synuclein representing as fluorescence in muscle cells (Figure 5A). The remarkable reduction of α-synuclein accumulation was observed in NL5901 treated with 1, 5, 10 μM FA (Figure 5A). Doses ranging from 1–10 μM FA showed a significant reduction of MFI by 20–30% (*p* < 0.05) compared with control DMSO-cultured worms (Figure 5B). However, in all doses of Rg3 treated groups, the MFI of YFP expression slightly decreased with no significant difference from the control group (Figure 5A,C).

### 2.5. FA and Rg3 Increase the Lifespan of 6-OHDA-Induced C. elegans

**Figure 6:**
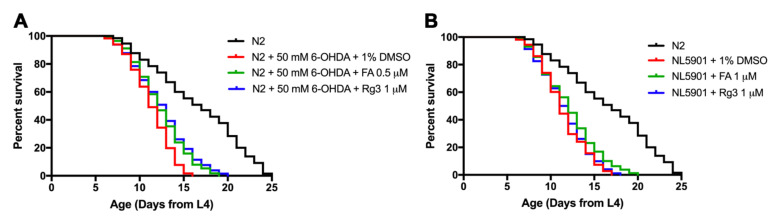
Effects of FA and Rg3 on lifespan of 6-OHDA-induced and transgenic α-synuclein NL5901 worms. (**A**) Survival curves of wild-type N2, 6-OHDA-induced, and FA/Rg3-treated worms. (**B**) Survival curves of wild-type N2, DMSO-cultured NL5901 and FA/Rg3-treated NL5901. The experiment was performed independently at least three times (number of worms = 30–40 animals/group per replicate).

Neurodegenerative diseases are usually associated with a shortened life expectancy [19]. Consistent with prior reports, our study showed that 6-OHDA neurotoxin significantly shortened *C. elegans* lifespan by 31.37% compared with normal worms (Figure 6A, Table 1). The treatment of 0.5 μM FA in 6-OHDA-induced worms significantly extended their lifespan by 6.22% compared with 6-OHDA/DMSO-treated worms (Figure 6A, Table 1). Likewise, Rg3 at 1 μM significantly increased the lifespan by 6.97% compared with 6-OHDA/DMSO-treated worms (*p* < 0.05) (Figure 6A, Table 1). 

### 2.6. FA Rescues Lifespan Shortened by α-Synuclein Overexpressed C. elegans

Since the previous study revealed that the transgenic α-synuclein-expressed worms have shortened lifespan [20], we tested the effect of FA and Rg3 on lifespan in transgenic NL5901 worms. The mean lifespan of N2 worms at 22 °C was approximately 16.49 ± 0.49 days. In NL5901 worms, the survival rate was significantly shortened by 31.05% compared with wild-type N2 worms (Figure 6B, Table 2). When NL5901 worms were treated with 0.5 μM FA, there was a significant increase of mean lifespan by 6.58%, compared with control DMSO-treated NL5901 group (Figure 6B, Table 2). However, treatment with 1 μM Rg3 slightly increased the mean lifespan by 1.29% with no significant difference from the control group.

### 2.7. FA and Rg3 Suppress the Apoptosis Regulators, egl-1/BH-3 and ced-3/caspase-9, and Increase sod-3 in 6-OHDA-Induced C. elegans

**Figure 7:**
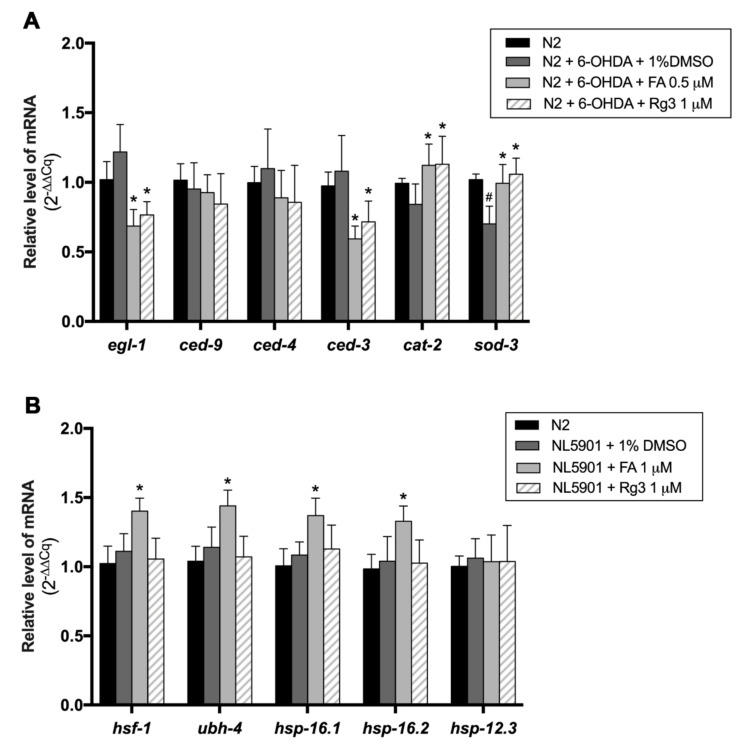
(**A**) Fold change of gene expression levels (2^−(ΔΔCq)^) of apoptosis genes in N2 worms treated with 6-OHDA, 6-OHDA/FA and 6-OHDA/Rg3. (**B**) Fold change of gene expression levels of protein degradation regulator genes in NL5901 worms treated with FA and Rg3. All targeted genes were measured in triplicate, and three independent biological triplicates were performed in each condition. The hash (#) indicates a significant difference between 6-OHDA-induced and uninduced worms (*p* < 0.05). The asterisk (*) indicates a significant difference between the control worms and the FA or Rg3-treated worms (*p* < 0.05).

Previous studies showed that apoptotic cell death accompanied DAergic neuronal loss in PD [21], hence we sought to determine whether FA and Rg3 rescued the DAergic neuron loss through suppressing the apoptosis signaling pathway. So, we examined the mRNA expression of apoptotic genes in 6-OHDA-induced and FA- or Rg3-treated worms. Exposure to 6-OHDA caused the slight increases in *egl-1* (orthologue of BH3), *ced-4* (orthologue of Apaf-1) and *ced-3* (orthologue of caspase-9) mRNA expression with no significant difference. However, both 0.5 μM FA and 1 μM Rg3 treatment could significantly reduce the *egl-1* and *ced-3* mRNA expression, which are proapoptotic genes, compared with control 6-OHDA-induced worms (*p* < 0.05) (Figure 7A). The expression of the free radical scavenging gene, *sod-3*, was significantly suppressed in 6-OHDA-induced worms. Interestingly, the expression of *sod-3* gene was significantly upregulated after treatment with 0.5 μM FA and 1 μM Rg3 (Figure 7A). Moreover, the *cat-2* gene, a gene encoding tyrosine hydroxylase for dopamine synthesis, was also significantly upregulated after treatment with 0.5 μM FA and 1 μM Rg3 (*p* < 0.05) (Figure 7A).

### 2.8. FA Enhances Protein Degradation Regulators, hsf-1, ubh-4, hsp-16.1 and hsp-16.2, in α-Synuclein-Overexpressed C. elegans Model

Previous reports demonstrated that α-synuclein aggregation is related to the dysfunction of the protein degradation pathway, including heat shock proteins (HSPs) and the ubiquitin proteasome system (UPS) [22]. To evaluate the underlying mechanisms of FA and Rg3 on the protein degradation mechanism, the mRNA levels of *hsf-1*, *ubh-4*, *hsp-16.1*, *hsp-16.2*, and *hsp-12.3*, which are associated with UPS and HSP in transgenic α-synuclein *C. elegans* were quantified by qRT-PCR. The results revealed that all degradation regulator genes were slightly increased in the transgenic α-synuclein-expressed worms but not significantly different as compared with wild-type N2 worms (Figure 7B). Interestingly, the levels of *hsf-1*, *ubh-4*, *hsp-16.1* and *hsp-16.2* mRNA of α-synuclein-expressed worms were significantly upregulated following treatment with 1 μM FA compared with control worms (*p* < 0.05), while treatment with 1 μM Rg3 showed no significant difference (Figure 7B).

## 3. Discussion

In this study, we investigated the anti-Parkinson activity of FA and Rg3 which are triterpene glycosides isolated from sea cucumber *C. frondosa* and plant *P. ginseng*, respectively. Our results have disclosed the neuroprotective effects of both FA (at 0.1, and 0.5 µM) and Rg3 (at 1, 5 and 10 µM) against 6-OHDA-induced neurodegeneration in *C. elegans* as revealed by the attenuation of DAergic neurodegeneration, improvement of basal slowing response behavior and extension of lifespan. Moreover, only FA (at 1, 5 and 10 µM) could reduce α-synuclein aggregation and extend lifespan in transgenic α-synuclein-expressed *C. elegans* model of PD. 

Our results revealed the neuroprotective effects of FA and Rg3 through the restored viability of DAergic neurons in 6-OHDA-induced *C. elegans* with the improvement of basal slowing response which is controlled by DAergic neurons [23] and the attenuation of the 6-OHDA-induced shortened lifespan [20,24,25]. Neurodegeneration induced by 6-OHDA leads to neuronal cell death via the overproduction of reactive oxygen species (ROS) and oxidative stress aggravating apoptosis [26]. In *C. elegans*, when apoptosis is activated, the EGL-1 (BH3 only protein) proapoptotic protein binds to CED-9 (Bcl-2 like antiapoptotic protein), thereby abolishing its inhibitory effect on CED-4 (Apaf-1-like adaptor protein). Then, CED-4 activates CED-3 (caspase), leading to cell death [27]. Our results revealed that the expression level of apoptosis regulators was not significantly different between normal N2 and 6-OHDA-induced worms, similar to a previous study suggesting that 6-OHDA mediates DAergic neurodegeneration in *C. elegans* independently from CED-4/CED-3 [28]. However, FA and Rg3 treatment significantly downregulated *egl-1* and *ced-3* in 6-OHDA-induced worms, indicating the antiapoptotic property of these compounds against 6-OHDA-induced neurodegeneration in *C. elegans*. In agreement with our finding, several studies showed the effect of Rg3 on apoptosis inhibition, including in neuronal cells [29,30], human endothelial cells by increasing BAX expression, while decreasing Bcl- 2 expression [31]. Moreover, Rg3 exerted a cardioprotective effect on myocardial injury by inhibiting caspase activation and apoptosis [32]. Evidence of the antiapoptotic effects of FA is limited, particularly against neuronal cell death. Our study firstly provided the evidence about the antiapoptotic activity of FA against 6-OHDA-induced neurodegeneration. Our study also found that FA and Rg3 could increase the expression of the free-radical scavenging gene (*sod-3*) which encodes the superoxide dismutase antioxidant enzyme for protecting cells against oxidative damage [33]. The activation of the *sod-3* gene has been shown to be crucially related to the neuroprotective effect against PD in the animal model [34,35]. Moreover, FA and Rg3 treatment could increase the *cat-2*, tyrosine hydroxylase, gene expression which controls dopamine synthesis. As shown in previous studies, both FA and Rg3 have antioxidative properties [36], for example, the ROS reduction in the transgenic *C. elegans* AD model by FA [16] and activations of CAT and SOD in cyclophosphamide-induced oxidative stress in the mice model by Rg3 [37]. Therefore, the effects of FA and Rg3 on the recovery of DAergic neurons in 6-OHDA-induced *C. elegans* may be associated with both antiapoptotic and antioxidative activities. The improvement of food-sensing behavior by FA and Rg3 treatment might be a result of this recovery of DAergic neurons together with the upregulation of the *cat-2* gene encoding enzyme for dopamine synthesis upon treatment. 

Since abnormal aggregation of α-synuclein is considered as major driver of PD progression, much ongoing research is exploring therapeutic strategies combatting α-synuclein aggregation and its toxicity [38]. The effectiveness of several triterpene ginsenosides, such as ginsenoside Rg1 and Rb1, on the diminution of α-synuclein aggregation has been intensively demonstrated [39,40,41]. Interestingly, the protective effect of Rg3 against proteotoxicity has been shown only in Alzheimer’s disease. Rg3 could decrease accumulation of amyloid β peptides (Aβ40 and Aβ42) by activating the degradation pathway in the cellular model of AD [42]. However, the inhibitory effects of plant-derived ginsenoside Rg3 and marine-derived FA on α-synuclein aggregation in PD have been rarely studied. Therefore, we herein investigated the effects of these compounds against α-synuclein aggregation in transgenic *C. elegans* model of PD. In the present study, we found a significant reduction of α-synuclein aggregation in transgenic *C. elegans* treated with FA. A previous study has reported significantly shortened lifespans in several strains of worms overexpressing α-synuclein [43]. Bioactive compound supplementation or genetic modification inhibiting α-synuclein aggregation have been shown to improve the longevity of transgenic *C. elegans* expressing human α-synuclein [20,44,45,46]. In this study, FA could extend the lifespan of the NL5901 worm which may be a consequence after reducing α-synuclein aggregates upon FA treatment. 

The dysfunction of the protein degradation pathway allows the abnormal aggregation of α-synuclein to persist and subsequently causes neurodegeneration in PD pathogenesis [38]. Since then, promoting the clearance of aggregated α-synuclein has become one of neuroprotective approaches for reducing neurotoxicity caused by α-synuclein [38,47]. The downregulation of proteasome subunits and the decrease of proteasome activities observed in the substantia nigra of PD patients have been shown to support an association of UPS impairment in PD pathogenesis [2,48]. In our study, we found that FA (at 1 µM) significantly upregulates the UPS-regulated gene, a ubiquitin carboxyl terminal hydrolase (*ubh-4*), which is an ortholog of human deubiquitinating UCHL5 enzyme required for maintaining proteostasis. A previous study revealed that a restored UCHL-5 level has a protective effect on decreasing α-synuclein aggregation and inhibiting neuronal cell death in an obese rat model [49]. Moreover, we also observed significant upregulation of *hsf-1*, *hsp-16.1* and *hsp-16.2* in the NL5901 worms upon FA treatment. This result suggested that *hsf-1, hsp-16.1* and *hsp-16.2* may be involved in the suppressing effect of FA against α-synuclein aggregation. However, the expression level of *hsp 12.3* did not alter upon FA treatment, indicating that it is not required for anti-α-synuclein aggregation activity of FA. HSPs are molecular chaperones responsible for the proper folding of α-synuclein and targeting misfolded α-synuclein for degradation [38]. A previous study by Liangliang et al., 2010, reported that constitutively active HSF-1 decreased the level of α-synuclein and reduced α-synuclein-induced cytotoxicity in SH-SY5Y cells [50]. HSF-1 is major transcription factor that activates the expression of many HSPs in response to proteotoxic stress [51]. A recent study showed that HSP16.2 could suppress Aβ toxicity by blocking its oligomerization, subsequently preventing Aβ-induced paralysis in transgenic *C. elegans* [52]. The activation of *hsp16.1* and *hsp16.2* genes by saponin derived from *Mormodica* exhibited antistress and antiaging properties [53]. To decrease α-synuclein aggregation, FA may activate the HSF-1 transcription factor, thereby activating it downstream, such as the *hsp16.1* and *hsp16*.2 genes we detected in the present study. In FA treatment, activation of the indicated HSPs may facilitate the delivery of α-synuclein to the proteasome and/or prevent misfolded α-synuclein from spontaneous aggregation. In turn, it results in an increasing level of α-synuclein available for UPS degradation. Taken together, our results have suggested that FA may protect against α-synuclein aggregation in transgenic *C. elegans* expressing α-synuclein, at least by activating the UPS pathway (*ubh-4*) and HSPs (*hsf-1*, *hsp-16.1* and *hsp-16.2*). However, further studies are required. For lifespan extension, the evidence that the upregulation of *hsp16.1* and *hsp16.2* affected the longevity of worms, as found by Lin et al., 2020 raises the possibility of a cellular event behind lifespan extension upon FA treatment. Increased lifespan may be a consequence of HSP activation or an indirect effect after the reduction of α-synuclein aggregation.

Unlike the FA treatment, a significant reduction of α-synuclein aggregates was not observed in the ginsenoside Rg3 treatment. Our results are in agreement with a previous study by Ardah et al., 2015, which reported that ginsenoside Rg3 did not exhibit significant inhibitory effects against α-synuclein aggregation and toxicity in a cellular model [41]. Correlating with the unchanged α-synuclein accumulation, Rg3 did not upregulate the expression of any genes in the protein degradation pathway. These findings have suggested that Rg3 have no effect on the inhibition of α-synuclein aggregation and lifespan extension in transgenic α-synuclein-expressed *C. elegans* model of PD.

Differences in anti-Parkinson activity between FA and Rg3 may be due to the differences in their chemical structures. The major difference between Rg3 and FA structures is the number of monosaccharide units connected to the aglycon part. Rg3 has two sugar rings at the C3 position of aglycone, while FA contains penta-saccharide-chain at the C3 position of aglycone. Several studies have demonstrated the structure−function relationships between the number and position of the sugar moiety of triterpene glycosides and their biological functions, such as in anti-amyloid β activity [54,55]. Moreover, FA contains a sulfate group at C4 of the first sugar ring of the glycoside residue and an acetyl group at the C16 position of aglycon residue [56]. The presence of a sulfate group and its impact on biological properties of triterpene glycosides has been reported [55]. Previous studies showed that cumariosides A_2_ and echinoside B, containing a sulfate group at C4 of their first sugar residue, exhibit stronger immunostimulatory and antifungal activity, respectively, than their desulfated derivatives [57,58]. Overall, our studies revealed that Rg3 only attenuates DAergic neurodegeneration, while FA protects against DAergic neurodegeneration and also diminishes α-synuclein aggregation in the *C. elegans* PD model. Based on previous reports about the structure−activity relationship of triterpene glycoside, we hypothesized that different anti-PD properties of FA and Rg3, particularly in reducing α-synuclein aggregation, may be due to either the number and/or the position of the saccharide unit, or acetyl or sulfated groups, or both. According to its wide spectrum of neuroprotective effects, in terms of recovery of neurodegeneration and reduction of α-synuclein aggregates, FA may be a promising therapeutic agent for treating PD. However, its cellular mechanism and structure−activity relationship against PD remain to be investigated.

## 4. Materials and Methods

### 4.1. Strains, Maintenance, and Synchronization

*C. elegans* wild-type N2, transgenic BZ555 (*dat-1*p::GFP; green fluorescent protein expression in DAergic neuronal soma and processes) and NL5901 (*unc-54*p::human α-synuclein::YFP+*unc-119*; yellow fluorescent protein expression in the muscles) strains used for the study were obtained from the *Caenorhabditis* Genetics Center (CGC, University of Minnesota, USA). All procedures performed in *C. elegans* were carried out according to protocols number MUSC60-049-398 approved by the MUSC-ACUC. Nematodes were maintained on nematode growth media (NGM) and fed with *Escherichia coli* OP50 strain as a food source at 20 °C by standard methods [59]. Synchronized eggs were isolated from adult worms by bleaching solution (12% NaClO and 10% 1 M NaOH) for 10 min, washed with M9 buffer twice, plated on NGM without bacteria, and incubated at 20 °C overnight to obtain newly hatched animals or L1 larvae.

### 4.2. Food Clearance Assay

Food clearance assay was performed to determine the effects of FA and Rg3 on *C. elegans* N2 feeding activity and toxicity [60]. FA and Rg3 were purchased from Sigma Aldrich (St. Louis, MO, USA). Both FA and Rg3 compounds were dissolved in DMSO to obtain various doses at 0.1, 0.5, 1, 5, 10, and 20 μM, with the final concentration of 1% (*v/v*) DMSO in treated cultures. OP50 *E. coli* were cultured in Luria−Bertani (LB) broth for 16 h, and then the optical density was adjusted at 600 nm (OD_600_) to 7.0 in S-medium (5.85 g NaCl, 1 g K_2_HPO_4_, 6 g KH_2_PO_4_, 1 mL cholesterol in ethanol, and distilled H_2_O up to 1L). Seventy-five microliters of the solution containing 60 μL of *E. coli* suspension, 10 μL of the compounds at various doses in S-medium, and 5 μL of S-medium containing 10 synchronized L1 N2 animals was incubated in a 96-well plate at 25 °C. The absorbance at 600 nm (OD_600_) was measured daily for 6 days using a versa max tunable microplate reader (Molecular Devices, Sunnyvale, CA, USA). The experiment was performed in triplicate independently.

### 4.3. Treatment of Worms with 6-OHDA, FA, and Rg3

Worms were treated with 6-OHDA (Sigma Aldrich, St. Louis, MO, USA) to selectively induce the degeneration of DAergic neurons [28]. The synchronized L3 larvae were incubated in a solution containing diluted OP50 mixed with 50 mM 6-OHDA and 10 mM ascorbic acid (Sigma Aldrich, St. Louis, MO, USA). The solution was mixed gently with a pipette every 10 min for 1 h at 20 °C. After induction, the worms were washed three times with M9 buffer and then transferred to OP50/NGM plates containing various doses at 0.1, 0.5, 1, 5, and 10 μM of FA and Rg3, and 50 μM 5-fluoro-2′-deoxyuridine (FUDR; Sigma Aldrich, St. Louis, MO, USA) that was used to suppress the new progeny production and treated for 72 h at 20 °C. In control experiments, the 6-OHDA-induced worms were incubated in OP50/NGM plates containing 1% (*v/v*) DMSO without the compounds (number of worms = 40–50 animals/group per replicate).

### 4.4. Quantitative Assay for DAergic Neurodegeneration

DAergic neurodegeneration was observed after 72 h treatment in OP50/6-OHDA/DMSO and OP50/6-OHDA/FA or Rg3 as described previously. The adult worms were washed three times with M9 buffer and then transferred onto 2% agarose pad on glass slides and anesthetized with a drop of 100 mM sodium azide (Sigma Aldrich, St. Louis, MO, USA) and finally enclosed by a slide cover glass. The immobilized worms were observed and photographed under a fluorescence microscope (BX53, Olympus Corp., Tokyo, Japan). Fluorescence intensity was measured by ImageJ software (National Institute of Health, NIH, Bethesda, ML, USA).

### 4.5. Analysis of the Basal Slowing Behavior

The basal slowing rate was investigated to test the basal slowing function controlled by DAergic neurons [18]. Typically, the worms move slowly on the bacterial lawn on the culture plates to consume bacterial food, compared with quick moving on plates without bacteria. N2 nematodes at L3 larvae stage were treated with either 6-OHDA+1% DMSO, or 6-OHDA+compounds (FA or Rg3), for 72 h at 20 °C, and then washed three times with M9 buffer to remove bacteria. The treated worms were transferred to the plates with or without bacterial supply and allowed to recover for 5 min. After that, body bending of all worms was recorded and counted in 10 s intervals. The numbers of worms body bending on the plates containing bacteria and without bacteria were measured and compared in each group. The experiment was performed independently at least three times (number of worms = 40–50 animals/group per replicate).

### 4.6. Quantitative Assay for α-Synuclein Accumulation

Analysis of α-synuclein accumulation was performed in the NL5901 strain treated with FA or Rg3 [43]. Synchronized L1 larvae were transferred onto the NGM plates containing various doses of FA, Rg3, and 150 mM FUDR and incubated for 72 h at 20 °C. Then, treated NL5901 worms were washed with M9 buffer, mounted onto agarose pad slides with 100 mM sodium azide, and enclosed with a slide cover glass. The fluorescence intensity of the accumulated α-synuclein in the worm muscles was assessed using a fluorescence microscope and quantified by using ImageJ software. The experiment was performed independently at least three times (number of worms = 40–50 animals/group per replicate).

### 4.7. Lifespan Assay

Wild-type synchronized L3 larvae were induced with 6-OHDA and transferred to OP50/NGM/FUDR plates containing FA (0.5 μM), or Rg3 (1 μM), or only DMSO. The numbers of live and dead worms were counted and recorded daily until all worms died, then the lifespan was calculated. The experiment was performed independently at least three times (number of worms = 30–40 animals/group per replicate).

### 4.8. Quantitative RT-PCR

Around 1000 worms in each treatment group were washed 3 times with dH_2_O and then collected as pellet worms. Briefly, total RNA was synthesized using the RNA extraction kit (Qiagen, Germany) following the manufacturer’s protocol. The RNA samples were stored at −80 °C until use. The RNA concentration was quantified using NanoDrop 2000 Spectrophotometer (Thermo Scientific, Waltham, MA, USA) [61]. For quantitative gene expression analyses, high capacity complementary DNA (cDNA) was generated from 2 µg of RNA, using the iScriptTMReverse Transcription Supermix for RT–qPCR (Bio-Rad, Hercules, CA, USA). Then, cDNA was diluted to a ratio of 1:10 with SsoFast EvaGreen Supermix with Low ROX qRT-PCR (Bio-Rad, Hercules, CA, USA) and mixed with primers of specific genes. Real-time PCR was then performed by the CFX96 Touch Real-time PCR detection system (Bio-Rad, Hercules, CA, USA). The amplification reaction was initiated by holding the sample at 95 °C for 30 s. Then, PCR samples were set for denaturing at 95 °C for 5 s and for annealing/extension at 60 °C for 30 s. After 44 cycles of repeat, the sample was then heated up to 95 °C for melt curve analysis, and the quantification cycle (Cq) values were obtained. All targeted genes were measured in triplicate, and at least three independent biological triplicates were detected in each condition. The Cq values were then calculated and compared via 2−(ΔΔCq) equation representing relative fold change in the expression of each gene with *act-1* as the internal control. The experiment was performed independently at least three times. The primers used for the qPCR were shown in the Table 3.

### 4.9. Statistical Analysis

All statistical analysis was determined by using GraphPad Prism software (GraphPad Software Inc., San Diego, CA, USA). Results were presented as mean ± SD and the differences between groups were compared using one-way ANOVA analysis following the Tukey−Kramer test for multiple comparison results. For grouped analyses, a two-way ANOVA series was used following Tukey’s multiple comparison for post hoc comparison. Survival plots were compared using the log-rank test. Probability levels (*p*-value) of < 0.05 were considered as minimum threshold for statistical significance. 

## 5. Conclusions

This novel study established that triterpene glycosides, FA from sea cucumber *C. frondosa* and Rg3 from plant *P. notoginseng*, promote neuroprotective effects against 6-OHDA-induced DAergic neurodegeneration. In addition, FA could also induce α-synuclein degradation in transgenic α-synuclein-expressed *C. elegans* via upregulation of *ubh-4*, *hsf-1*, *hsp-16.1* and *hsp-16.2* expression. Therefore, FA may be a potential candidate to be further developed as a therapeutic agent against PD pathogenesis on both DAergic neurodegeneration and α-synuclein aggregation.

## Figures and Tables

**Table 1:** Mean lifespan, maximum lifespan, percentage of increase lifespan and significant *p* values of 6-OHDA-induced N2 worms treated with FA and Rg3.

Treatment	Mean Lifespan (Day)	Maximum Lifespan	% Increase Lifespan	Significance (*p*-Value)
N2	16.49 ± 0.49	25		
N2 + 50 mM 6-OHDA + 1% DMSO	11.31 ± 0.95	16	−31.37 (compared with N2)	**** *p* < 0.0001
N2 + 50 mM 6-OHDA + 0.5 μM FA	12.34 ± 0.97	19	6.22 (compared with 6-OHDA + DMSO)	*** (*p* = 0.004)
N2 + 50 mM 6-OHDA + 1 μM Rg3	12.46 ± 0.54	20	6.97	**** *p* < 0.0001

**Table 2:** Mean lifespan, maximum lifespan, percentage of increased lifespan and significant *p* values of N2 and NL5901 worms treated with FA and Rg3.

Treatment	Mean Lifespan (Day)	Maximum Lifespan	% Increased Lifespan	Significance (*p*-Value)
N2	16.49 ± 0.49	25		
NL5901 + 1% DMSO	11.37 ± 0.80	20	−31.05 (compared with N2)	**** *p* < 0.0001
NL5901 + 1 μM FA	12.11 ± 0.88	23	6.58 (compared with NL5901 + 1% DMSO)	* (*p* = 0.011)
NL5901 + 1 μM Rg3	11.51 ± 0.96	24	1.29	Not significant (*p* = 0.477)

**Table 3:** Primer lists used in this study.

Foward (5′→3′)		Reverse (5′→3′)
**Apoptosis mediators**		
*egl-1*	CTAGCAGCAATGTGCGATGAC	GGAA GCATGGGCCGAGTAG
*ced-9*	TGCTCAGGACTTGCCATCAC	TTGACTCTCCGATGGACATTCTT
*ced-4*	AAGTCGAGGATTAGTCGGTGTTG	AGAGCCATTGCGAGTGACTTG
*ced-3*	TCAACGCGGCAAATGCT	GCCTGCACAAAAACGATTTTC
**Antioxidant mediators**		
*sod-3*	AGCATCATGCCACCTACGTGA	CACCACCATTGAATTTCAGCG
**Dopamine synthesis**		
*cat-2*	GCACGTTTTGAGTTGGGTCC	ATGCAGCCAGGATTGTCGAA
**Protein degradation mediators**		
*hsf-1*	ATGCAGCCAGGATTGTCGAA	GCACGTTTTGAGTTGGGTCC
*ubh-4*	GCACTTGTTCCAAACCGCAA	GACGTCGGCGATTGTTTTCC
*hsp-16.1*	GCAGAGGCTCTCCATCTGAA	GCTTGAACTGCGAGACATTG
*hsp-16.2*	GTCACTTTACCACTATTTCCGT	CAATCTCAGAAGACTCAGATGG
*hsp-12.3*	GCCATTCCAGAAAGGAGATG	CGTTTGGCAAGAAGTTGTGA
**Housekeeping gene**		
*act-1*	AGGTTGCCGCTCTTGTTGTA	CGTGGTCTTCCGACAATGGA

## Data Availability

The data supporting the conclusion in this study are available on request from the corresponding author.

## Sample Availability

Samples of the compounds are not available from the authors.

## References

[1] Moustafa A.A., Chakravarthy S., Phillips J.R., Gupta A., Keri S., Polner B., Frank M.J., Jahanshahi M. (2016). Motor symptoms in Parkinson’s disease: A unified framework. Neurosci. Biobehav. Rev..

[2] McNaught K.S.P., Jenner P. (2001). Proteasomal function is impaired in substantia nigra in Parkinson’s disease. Neurosci. Lett..

[3] Connolly B.S., Lang A.E. (2014). Pharmacological treatment of Parkinson disease: A review. JAMA.

[4] Alihosseini F., Sun G. (2016). Plant-based compounds for antimicrobial textiles. Antimicrobial Textiles.

[5] Lorent J.H., Quetin-Leclercq J., Mingeot-Leclercq M.-P. (2014). The amphiphilic nature of saponins and their effects on artificial and biological membranes and potential consequences for red blood and cancer cells. Org. Biomol. Chem..

[6] Fujihara K., Shimoyama T., Kawazu R., Sasaki H., Koyama K., Takahashi K., Kinoshita K. (2021). Amyloid β aggregation inhibitory activity of triterpene saponins from the cactus *Stenocereus pruinosus*. J. Nat. Med..

[7] Chen X.-C., Zhu Y.-G., Zhu L.-A., Huang C., Chen Y., Chen L.-M., Fang F., Zhou Y.-C., Zhao C.-H. (2003). Ginsenoside Rg1 attenuates dopamine-induced apoptosis in PC12 cells by suppressing oxidative stress. Eur. J. Pharmacol..

[8] Li X.-F., Lui C.N.-P., Jiang Z.-H., Ken Y.K.-L. (2011). Neuroprotective effects of ginsenosides Rh1 and Rg2 on neuronal cells. Chin. Med..

[9] Zhang Y., Yang X., Wang S., Song S. (2019). Ginsenoside Rg3 prevents cognitive impairment by improving mitochondrial dysfunction in the rat model of Alzheimer’s disease. J. Agric. Food Chem..

[10] Kim S., Kim T., Ahn K., Park W.-K., Nah S.-Y., Rhim H. (2004). Ginsenoside Rg3 antagonizes NMDA receptors through a glycine modulatory site in rat cultured hippocampal neurons. Biochem. Biophys. Res. Commun..

[11] Moon J.-H., Lee J.-H., Lee Y.-J., Park S.-Y. (2016). Autophagy flux induced by ginsenoside-Rg3 attenuates human prion protein-mediated neurotoxicity and mitochondrial dysfunction. Oncotarget.

[12] Li X., Roginsky A.B., Ding X.Z., Woodward C., Collin P., Newman R.A., Bell J.R.H., Adrian T.E. (2008). Review of the apoptosis pathways in pancreatic cancer and the anti-apoptotic effects of the novel sea cucumber compound, Frondoside A. Ann. N. Y. Acad. Sci..

[13] Ma X., Kundu N., Collin P.D., Goloubeva O., Fulton A.M. (2012). Frondoside A inhibits breast cancer metastasis and antagonizes prostaglandin E receptors EP4 and EP2. Breast Cancer Res. Treat..

[14] Jin J.-O., Shastina V.V., Shin S.-W., Xu Q., Park J.-I., Rasskazov V.A., Avilov S.A., Fedorov S.N., Stonik V.A., Kwak J.-Y. (2009). Differential effects of triterpene glycosides, frondoside A and cucumarioside A2-2 isolated from sea cucumbers on caspase activation and apoptosis of human leukemia cells. FEBS Lett..

[15] Attoub S., Arafat K., Gélaude A., Al Sultan M.A., Bracke M., Collin P., Takahashi T., Adrian T.E., De Wever O. (2013). Frondoside A suppressive effects on lung cancer survival, tumor growth, angiogenesis, invasion, and metastasis. PLoS ONE.

[16] Tangrodchanapong T., Sobhon P., Meemon K. (2020). Frondoside A attenuates amyloid-β proteotoxicity in transgenic *Caenorhabditis elegans* by suppressing its formation. Front. Pharmacol..

[17] Harrington A.J., Hamamichi S., Caldwell G.A., Caldwell K.A. (2010). *C. elegans* as a model organism to investigate molecular pathways involved with Parkinson’s disease. Dev. Dyn..

[18] Sawin E.R., Ranganathan R., Horvitz H.R. (2000). *C. elegans* locomotory rate is modulated by the environment through a dopaminergic pathway and by experience through a serotonergic pathway. Neuron.

[19] Ishihara L.S., Cheesbrough A., Brayne C., Schrag A. (2007). Estimated life expectancy of Parkinson’s patients compared with the UK population. J. Neurol. Neurosurg. Psychiatry.

[20] Malaiwong N., Chalorak P., Jattujan P., Manohong P., Niamnont N., Suphamungmee W., Sobhon P., Meemon K. (2019). Anti-Parkinson activity of bioactive substances extracted from *Holothuria leucospilota*. Biomed. Pharmacother..

[21] Venderova K., Park D.S. (2012). Programmed cell death in Parkinson’s disease. Cold Spring Harb. Perspect. Med..

[22] Jones D.R., Moussaud S., McLean P. (2014). Targeting heat shock proteins to modulate α-synuclein toxicity. Ther. Adv. Neurol. Disord..

[23] Cooper J.F., Dues D.J., Spielbauer K.K., Machiela E., Senchuk M.M., Van Raamsdonk J.M. (2015). Delaying aging is neuroprotective in Parkinson’s disease: A genetic analysis in *C. elegans* models. NPJ Parkinsons Dis..

[24] Chalorak P., Jattujan P., Nobsathian S., Poomtong T., Sobhon P., Meemon K. (2018). *Holothuria scabra* extracts exhibit anti-Parkinson potential in *C. elegans*: A model for anti-Parkinson testing. Nutr. Neurosci..

[25] Tsai R.-T., Tsai C.-W., Liu S.-P., Gao J.-X., Kuo Y.-H., Chao P.-M., Hung H.-S., Shyu W.-C., Lin S.-Z., Fu R.-H. (2020). Maackiain ameliorates 6-hydroxydopamine and SNCA pathologies by modulating the PINK1/Parkin pathway in models of Parkinson’s disease in *Caenorhabditis elegans* and the SH-SY5Y Cell Line. Int. J. Mol. Sci..

[26] Soto-Otero R., Méndez-Álvarez E., Hermida-Ameijeiras Á., Muñoz-Patiño A.M., Labandeira-Garcia J.L. (2000). Autoxidation and neurotoxicity of 6-hydroxydopamine in the presence of some antioxidants. J. Neurochem..

[27] Lettre G., Hengartner M.O. (2006). Developmental apoptosis in *C. elegans*: A complex CEDnario. Nat. Rev. Mol. Cell Biol..

[28] Nass R., Hall D.H., Miller D.M., Blakely R.D. (2002). Neurotoxin-induced degeneration of dopamine neurons in *Caenorhabditis elegans*. Proc. Natl. Acad. Sci. USA.

[29] Hou J., Xue J., Wang Z., Li W. (2018). Ginsenoside Rg3 and Rh2 protect trimethyltin-induced neurotoxicity via prevention on neuronal apoptosis and neuroinflammation. Phytother. Res..

[30] Kim J.-H., Cho S.Y., Lee J.-H., Jeong S.M., Yoon I.-S., Lee B.-H., Lee J.-H., Pyo M.K., Lee S.-M., Chung J.-M. (2007). Neuroprotective effects of ginsenoside Rg3 against homocysteine-induced excitotoxicity in rat hippocampus. Brain Res..

[31] Min J.-K., Kim J.-H., Cho Y.-L., Maeng Y.-S., Lee S.-J., Pyun B.-J., Kim Y.-M., Park J.H., Kwon Y.-G. (2006). 20(S)-Ginsenoside Rg3 prevents endothelial cell apoptosis via inhibition of a mitochondrial caspase pathway. Biochem. Biophys. Res. Commun..

[32] Wang Y., Hu Z., Sun B., Xu J., Jiang J., Luo M. (2015). Ginsenoside Rg3 attenuates myocardial ischemia/reperfusion injury via Akt/endothelial nitric oxide synthase signaling and the B-cell lymphoma/B-cell lymphoma-associated X protein pathway. Mol. Med. Report..

[33] Sheng Y., Abreu I.A., Cabelli D.E., Maroney M.J., Miller A.-F., Teixeira M., Valentine J.S. (2014). Superoxide dismutases and superoxide reductases. Chem. Rev..

[34] Peng J., Stevenson F.F., Doctrow S.R., Andersen J.K. (2005). Superoxide dismutase/catalase mimetics are neuroprotective against selective paraquat-mediated dopaminergic neuron death in the substantial nigra: Implications for Parkinson disease. J. Biol. Chem..

[35] Pong K., Doctrow S.R., Baudry M. (2000). Prevention of 1-methyl-4-phenylpyridinium- and 6-hydroxydopamine-induced nitration of tyrosine hydroxylase and neurotoxicity by EUK-134, a superoxide dismutase and catalase mimetic, in cultured dopaminergic neurons. Brain Res..

[36] Li G., Zhang X.X., Lin L., Liu X.N., Ma C.J., Li J., Wang C.B. (2014). Preparation of Ginsenoside Rg3 and protection against H_2_O_2_-induced oxidative stress in human neuroblastoma SK-N-SH cells. J. Chem..

[37] Wei X., Su F., Su X., Hu T., Hu S. (2012). Stereospecific antioxidant effects of ginsenoside Rg3 on oxidative stress induced by cyclophosphamide in mice. Fitoterapia.

[38] Zhang G., Xia Y., Wan F., Ma K., Guo X., Kou L., Yin S., Han C., Liu L., Huang J. (2018). New perspectives on roles of alpha-synuclein in Parkinson’s disease. Front. Aging Neurosci..

[39] Van Kampen J.M., Baranowski D.B., Shaw C.A., Kay D.G. (2014). Panax ginseng is neuroprotective in a novel progressive model of Parkinson’s disease. Exp. Gerontol..

[40] Heng Y., Zhang Q.-S., Mu Z., Hu J.-F., Yuan Y.-H., Chen N.-H. (2016). Ginsenoside Rg1 attenuates motor impairment and neuroinflammation in the MPTP-probenecid-induced parkinsonism mouse model by targeting α-synuclein abnormalities in the substantia nigra. Toxicol. Lett..

[41] Ardah M.T., Paleologou K.E., Lv G., Menon S.A., Abul Khair S.B., Lu J.-H., Safieh-Garabedian B., Al-Hayani A.A., Eliezer D., Li M. (2015). Ginsenoside Rb1 inhibits fibrillation and toxicity of alpha-synuclein and disaggregates preformed fibrils. Neurobiol. Dis..

[42] Yang L., Hao J., Zhang J., Xia W., Dong X., Hu X., Kong F., Cui X. (2009). Ginsenoside Rg3 promotes beta-amyloid peptide degradation by enhancing gene expression of neprilysin. J. Pharm. Pharmacol..

[43] Bodhicharla R., Nagarajan A., Winter J., Adenle A., Nazir A., Brady D., Vere K., Richens J., O’Shea P., Bell D.R. (2012). Effects of α-synuclein overexpression in transgenic *Caenorhabditis elegans* strains. CNS Neurol. Disord. Drug Targets.

[44] Yang X., Zhang M., Dai Y., Sun Y., Aman Y., Xu Y., Yu P., Zheng Y., Yang J., Zhu X. (2020). Spermidine inhibits neurodegeneration and delays aging via the PINK1-PDR1-dependent mitophagy pathway in C. elegans. Aging.

[45] Virendra S., Suresh C.P., Deepti Y., Sudeep T., Supinder K., Gupta M.M., Aamir N., Rakesh P. (2012). Iridoid compound 10-O-trans-p-coumaroylcatalpol extends longevity and reduces alpha synuclein aggregation in *Caenorhabditis elegans*. CNS Neurol. Disord. Drug Targets.

[46] Kaur S., Nazir A. (2015). Potential role of protein stabilizers in amelioration of Parkinson’s disease and associated effects in transgenic *Caenorhabditis elegans* model expressing alpha-synuclein. RSC Adv..

[47] Esposito L.A., Hopkins C.R. (2016). Targeting α-synuclein as a Parkinson’s disease therapeutic. Novel Therapeutic Approaches to the Treatment of Parkinson’s Disease: An Overview and Update.

[48] Grünblatt E., Mandel S., Jacob-Hirsch J., Zeligson S., Amariglo N., Rechavi G., Li J., Ravid R., Roggendorf W., Riederer P. (2004). Gene expression profiling of parkinsonian substantia nigra pars compacta; alterations in ubiquitin-proteasome, heat shock protein, iron and oxidative stress regulated proteins, cell adhesion/cellular matrix and vesicle trafficking genes. J. Neural Transm..

[49] Shruthi K., Reddy S.S., Reddy P.Y., Shivalingam P., Harishankar N., Reddy G.B. (2016). Amelioration of neuronal cell death in a spontaneous obese rat model by dietary restriction through modulation of ubiquitin proteasome system. J. Nutr. Biochem..

[50] Liangliang X., Yonghui H., Shunmei E., Shoufang G., Wei Z., Jiangying Z. (2010). Dominant-positive HSF1 decreases alpha-synuclein level and alpha-synuclein-induced toxicity. Mol. Biol. Rep..

[51] Fujikake N., Nagai Y., Popiel H.A., Okamoto Y., Yamaguchi M., Toda T. (2008). Heat shock transcription factor 1-activating compounds suppress polyglutamine-induced neurodegeneration through induction of multiple molecular chaperones. J. Biol. Chem..

[52] Fonte V., Kipp D.R., Yerg J., Merin D., Forrestal M., Wagner E., Roberts C.M., Link C.D. (2008). Suppression of in vivo β-amyloid peptide toxicity by overexpression of the HSP-16.2 small chaperone protein. J. Biol. Chem..

[53] Lin C., Chen Y., Lin Y., Wang X., Hu L., Cao Y., Chen Y. (2021). Antistress and anti-aging activities of *Caenorhabditis elegans* were enhanced by Momordica saponin extract. Eur. J. Nutr..

[54] Zhang M., Qian F., Liu Q., Qian C., Thu P.M., Wang Y., Zheng Z.-G., Yang H., Li P., Xu X. (2017). Evaluation of structure–activity relationships of ginsenosides against amyloid β induced pathological behaviours in transgenic *Caenorhabditis elegans*. RSC Adv..

[55] Kim S.K., Himaya S.W.A. (2012). Triterpene Glycosides from Sea Cucumbers and Their Biological Activities. Adv. Food Nutr. Res..

[56] Park J.-I., Bae H.-R., Kim C.G., Stonik V.A., Kwak J.-Y. (2014). Relationships between chemical structures and functions of triterpene glycosides isolated from sea cucumbers. Front. Chem..

[57] Aminin D.L., Silchenko A.S., Avilov S.A., Stepanov V.G., Kalinin V.I. (2010). Immunomodulatory action of monosulfated triterpene glycosides from the sea cucumber Cucumaria okhotensis: Stimulation of activity of mouse peritoneal macrophages. Nat. Prod. Commun..

[58] Kitagawa I., Kobayashi M., Inamoto T., Fuchida M., Kyogoku Y. (1985). Marine natural products. XIV. Structures of Echinosides A and B, antifungal lanostane-oligosides from the sea cucumber *Actinopyga echinites* (Jaeger). Chem. Pharm. Bull..

[59] Brenner S. (1974). The genetics of *Caenorhabditis elegans*. Genetics.

[60] Gomez-Amaro R.L., Valentine E.R., Carretero M., LeBoeuf S.E., Rangaraju S., Broaddus C.D., Solis G.M., Williamson J.R., Petrascheck M. (2015). Measuring food intake and nutrient absorption in *Caenorhabditis elegans*. Genetics.

[61] Chalorak P., Dharmasaroja P., Meemon K. (2020). Downregulation of eEF1A/EFT3-4 enhances dopaminergic neurodegeneration after 6-OHDA exposure in *C. elegans* model. Front. Neurosci..

